# Pre-operative body shape concerns moderate excess weight loss trajectory in bariatric surgery patients: a 2-year longitudinal study

**DOI:** 10.1007/s40519-024-01660-w

**Published:** 2024-04-24

**Authors:** Oriana Moro, Umberto Albert, Elide Francesca De Caro, Silvia Palmisano, Manuela Mastronardi, Lisa Di Blas

**Affiliations:** 1 Department of Life Sciences, University of Trieste, Trieste, Italy; 2 Department of Mental Health, ASUGI, Trieste, Italy; 3 Department of Medicine, Surgery and Health Sciences, University of Trieste, Trieste, Italy

**Keywords:** Body dissatisfaction, Body Shape Questionnaire, Bariatric surgery psychology, Post-operative weight loss trend, Pathoplasty model

## Abstract

**Purpose:**

The main research aim was to inspect whether pre-operative body shape concerns and discomfort as Body Shape Questionnaire (BSQ) scores moderate post-operative weight loss trajectory in bariatric patients.

**Methods:**

Two studies were conducted. Study 1 analyzed cross-sectional data and verified the structural validity of the 34-item BSQ questionnaire on a sample of 327 candidates for bariatric surgery. Study 2 examined longitudinal data, with objective Body Mass Index (BMI) recorded every 6 months, from surgery intervention on, with 5 measurement occasions, from 111 patients who initially completed BSQ as bariatric surgery candidates and then underwent periodic medical post-operative follow-ups, over 2 years.

**Results:**

In Study 1, confirmatory factor analysis of a single-dimension model yielded acceptable fit indices and high internal consistency levels. Study 2 showed that post-operative excess BMI reduction trend was not linear and pre-operative BSQ scores moderated it, with a higher risk of weight regain in patients who initially were less concerned with their body shape.

**Conclusions:**

The present findings support the structural validity of the BSQ questionnaire in bariatric candidates and call attention on the role of pre-operative body shape concerns on post-operative weight loss trajectories over 2 years, in accordance with a pathoplasty model. They suggest the need for systematic attention on perceived body image and psychological paths aimed to help bariatric patients regain positive attitudes towards their own body.

*Level of evidence* III, well-designed cohort

## Introduction

Obesity represents a health risk condition, widespread enough to be regarded as epidemic in Western societies [1]. In Italy, its incidence is equal to 12% of the population, while 34% is overweight [2]. Bariatric surgery currently is the most effective treatment for people suffering from severe obesity [3], further effectively reducing cardio-vascular risks and all-cause mortality [3–5]. Surgery, however, does not guarantee that bariatric patients reach and maintain a healthy weight over time. Indeed, post-bariatric weight loss depends on several pre- and post-operative factors, which include medical conditions, eating and physical habits, psychopathological profiles, interpersonal relationships, and body image [6–12].

Trajectories of post-operative weight loss generally reveal that bariatric patients rapidly lose weight during the first semester and reach the maximum percentage of excess weight loss between 12 and 18 months after intervention; weight reduction gradually stagnates during the second year and risk for weight regain increases between 3 and 7 years after intervention [13, 14]. Although trajectories may differ among individuals [15, 16], weight loss at 6 months is a valid predictor of bariatric success on the long run [15], in addition to pre-operative body mass index [17], which generally predicts post-intervention weight loss across short- to medium-time intervals, with higher initial BMI predicting lower bariatric success [6, 18].

Research on effects of pre-operative psychological factors has also yielded relevant findings in relation to surgical bariatric intervention, though empirical results are mixed. In fact, people suffering from obesity generally report a lower quality of life, higher stigmatization, higher psychological as well as psychiatric disturbances, worse body images, lower self-esteem, more eating disordered habits, and all these individual domains tend to improve after bariatric surgery, though leaving scars [3, 10, 11, 19]. Nevertheless, there still need for empirical research on their effects on bariatric surgery outcome, because current findings tend to be not fully consistent in relation to how these pre-operative risk factors help predict post-intervention outcome [6, 7, 11, 20]. In the current study, we focused our attention on individual attitudes towards body appearance, that is, a basic component of the higher order construct of body image, which also includes body perception and body-related behaviors [21, 22]. Specifically, the attitudinal domain of body image often is overlapped with body dissatisfaction, but it rather represents feelings and evaluations of our own body shape, attractiveness and weight, including dissatisfaction, discomfort and concerns with body appearance as well as importance attached to physical appearance [21–23], and it has been extensively demonstrated to represent a risk factor for the development, maintenance and exacerbation of weight- and eating-disordered conditions, in Western countries [24, 25].

The attitudinal domain of body image usually is assessed among candidates for bariatric surgery [26, 27]. Indeed, empirical research indicates that individuals with obesity, women especially [28], refer moderate to marked concerns with their body shape compared to population in the range of normal weight [8, 29, 30], but there is necessarily not a linear relationship between BMI and body dissatisfaction among people suffering from obesity [31]. Furthermore, discomfort with body shape may underlie motivation to undertake a surgical treatment [29, 32]. Accordingly, bariatric surgery has also been demonstrated to favor an overall improvement in body satisfaction and in psychological well-being, with post-operative decreases in weight also correlating with post-operative improvements in body image, although bariatric patients still suffer from a higher dissatisfaction with their own body image compared to normative population [26, 30, 31, 33, 34].

Less consistent findings emerge from longitudinal studies aimed at inspecting how pre-operative body dissatisfaction helps predict post-intervention weight loss months or years later. Hrabosky and colleagues [31], for example, reported that individual differences in pre-surgery body dissatisfaction did not predict post-operative BMI loss at 6 or 12 months, but findings by Agüera et al. [6] indicate a link across time. Furthermore, some studies suggest that initial body dissatisfaction has an indirect effect on weight reduction by favoring post-operative treatment adherence, including dietary and exercising prescriptions [35, 36], and that the vulnerability effect of pre-intervention psychopathological characteristics might emerge later, when the surgical intervention itself does favor the initial quick weight reduction no longer [37]. Less attention has been put on how psychological variables shape post-surgical weight loss trajectories, in accordance with a pathoplasty model, which links relatively stable individual psychological differences to the course of the illness and recovery [38]. For example, Oltmanns et al. [37] found that stress and anxiety moderate weight reduction trends over 5 years. To our knowledge, no similar empirical results are available for individual differences in body dissatisfaction.

Research findings are not fully consistent also due to an inhomogeneity in body dissatisfaction assessment instruments [9]. The BSQ questionnaire currently is among the most widely applied tools for body dissatisfaction in patients with severe obesity [26], being further recommended in Italy as a preliminary screening tool for candidates for bariatric surgery interventions [27]. Despite its relevance in bariatric obesity, no empirical study has examined the validity of BSQ in candidates for bariatric surgery yet.

Post-intervention indices of weight loss are homogeneous neither. Dietel and colleagues [39] recommended percentage of excess BMI loss (%EBMIL) as the best method for comparing obesity treatments results; this quantitative index evaluates a patient’s BMI loss against a pre-set optimal or expected BMI of 25, for any bariatric patient, and a value equal to 50% is regarded as a minimum threshold for a successful post-operative outcome. Alternatively, percentage of weight loss against an expected weight loss (%EWL) has also been proposed, and it generally parallels %EBMIL. Nevertheless, the %EBMIL index has been questioned because sensitive to pre-surgery BMI levels: Bariatric patients with pre-operative BMI < 50 generally meet the post-operative threshold value of 50% more easily than patients with BMI ≥ 50. Therefore, Baltazar and colleagues [40] suggested to calculate the %EBMIL after adjusting a patient’s expected BMI on the basis of their pre-surgery BMI. Finally, percentage of total weight loss (%TWL) has also been recommended as the preferable metric choice, because less linked to pre-surgery BMI, with values lower than 20% indicating suboptimal weight loss [41]. In the current study, we examined weight loss against three indices, i.e., %EBMIL, adjusted %EBMIL, and %TWL.

*Research aims* The present research project was focused on the Body Shape Questionnaire [42, 43] as an overall measure of feelings of fatness and discomfort and concerns with body shape. The main objective of our research was to inspect how a patient’s pre-intervention body shape concerns as BSQ scores moderates the overall post-operative weight loss trajectory, over 2 years. To this aim, we preliminarily examined cross-sectional data to test the structural validity of the BSQ questionnaire in candidates for bariatric surgery (Study 1), and then examined longitudinal data from patients who underwent bariatric surgery to describe their post-surgery weight loss trajectory, that is, shape of individual changes over four follow-ups, in function of their pre-surgery weight and body shape dissatisfaction (Study 2). Secondary aims were to provide BSQ normative scores for candidates for bariatric surgical intervention (Study 1), and to inspect how pre-intervention BSQ levels predict weight changes (operationalized as %TWL, %EBMIL, and adjusted %EBMIL) from intervention to a given post-operative time point, i.e., 6, 12, 18, and 24 months later.

## Methods

### Study 1

#### Data

The present retrospective monocentric study used self-reports from 359 (68% females) candidates for obesity surgery, at the Bariatric Surgery Center of the Trieste] University Hospital, between 2008 and 2022; their average age was 42.6 ± 10.9 years, with no gender differences, and their average objective BMI was 42.6 ± 6.4, with a marginal gender difference (∆_M–F_ = 2.1, *p* < 0.01, η^2^ = 0.02). Patients suffering from obesity become candidates for bariatric surgery after experiencing failure of non-surgical treatments, are aged ≥ 18 years, their BMI generally is ≥ 35, with or without obesity-related comorbidities, and are patients with acceptable operative risks. No exclusion criteria of interviewed candidates were applied for the present data set.

#### Materials and procedure

At the Bariatric Surgery Center of the Trieste University Hospital, candidates to bariatric surgery are assessed by a team of specialists, including bariatric surgeons, gastroenterologists, dieticians, psychiatrists, and psychologists; moreover, candidates provide self-reports on standardized tools aimed at evaluating their profiles in relation to mental health, eating-related disturbances, and body attitudes. In the current study, we examined retrospective data on self-reported body concerns and discomfort.

*Body Shape Questionnaire (BSQ)* The Body Shape Questionnaire [BSQ; 42] was routinely administered to candidates for bariatric treatments, at the Trieste University Hospital, to systematically assess their pre-surgery degree of body dissatisfaction, concerns, and distress about their own body shape, particularly related to feeling fat. Scores can range from 34 to 204: scores in the range of 80–110 suggest mild concerns and discontent, 111–140 a moderate level, 141 and above a marked concerns and dissatisfaction [42]. For this sample, McDonald’s omega value of internal consistency was equal to 0.96, with item-scale correlations ranging between 0.25 and 0.84.

Actual weight as BMI was also recorded together with a candidate’s self-reported ideal target weight to reach after intervention.

#### Analysis

Confirmatory factor analysis (Jamovi 2.3.24 solid package) was applied to the data set of self-reports on the 34 BSQ items and a single-factor model was tested, also against different factor structures; the Comparative Fit Index (CFI > 0.90), Tucker–Lewis Index (TLI > 0.90), root mean square error of approximation (RMSEA < 0.08), and standardized root mean square residual (SRMR < 0.08) indicated the goodness of model fit; the residual correlation matrix among the items was inspected to improve the factor solution.

### Study 2

#### Data

This longitudinal study included data from a subsample of the Study 1 candidates, i.e., 111 adult patients (36 males, 32.4%, and 75 females, 67.6%) who underwent bariatric surgical intervention, between 2012 and 2019, at the same Bariatric Surgery Center, and satisfied two inclusion criteria, i.e., a BMI ≤ 60 and regular post-surgery medical check-ups, every 6 months, over a period of 2 years.

Candidates to bariatric surgery are not eligible for intervention if they present severe mental and psychological disorders, substance use disorders, and medical conditions precluding probable survival from surgery. Before surgical intervention, all patients are informed on surgical technique and risks.

#### Measures

*Independent variables* Patients’ BSQ scores and self-reported ideal target weight (also transformed into self-reported ideal BMI) collected at interview for eligibility to bariatric intervention (Study 1) were predictors, together with actual BMI at intervention; gender, age, and type of intervention were also taken under control, i.e., bypass gastrectomy (a surgical technique in which the stomach is divided into a small gastric pouch and completely separated from the bypassed stomach) and sleeve gastrectomy (which provides about 75% gastric reduction and the stomach is reshaped like a sleeve).

*Outcome* Objective BMI was recorded at each post-operative medical follow-up, every 6 months, i.e., 4 post-operative BMI records, and the outcome of bariatric intervention was calculated as percentage of total weight loss (%TWL), percentage of excess BMI loss (%EBMIL) and percentage of adjusted excess BMI loss (adjusted %EBMIL) [39–41], with the indices calculated as follows:

$$\mathrm{\%TWL }= (({{\text{Kg}}}_{{\text{Preoperative}}} - {{\text{Kg}}}_{{\text{Postoperative}}}) / {{\text{Kg}}}_{{\text{Preoperative}}}))*100$$

$$\%{\text{EBMIL}} = (({{\text{BMI}}}_{{\text{Preoperative}}}-{{\text{BMI}}}_{{\text{Postoperative}}} ) / ({{\text{BMI}}}_{{\text{Preoperative}}} -25))*100$$

$${\text{Adjusted}} \%{\text{EBMIL}} = (({{\text{BMI}}}_{{\text{Preoperative}}}-{{\text{BMI}}}_{{\text{Postoperative}}} ) / ({{\text{BMI}} }_{{\text{Preoperative}}} -{\text{PBMI}}))*100$$

where the expected BMI value is equal to a pre-set BMI of 25 for each patient when the traditional %EBMIL index is calculated, but corresponds to a personal BMI (PBMI) when the adjusted %EBMIL is calculated, with PBMI = BMI_Preoperative_ × 0.4 + 11.75; BMI_Postoperative_ corresponds to actual BMI at a given post-operative occasion. A value of 50 represents a threshold for post-operative success, i.e., there has been a loss of BMI in excess equal to at least half of the expected loss against an optimal BMI, while a value ≥ 100 shows that the post-operative weight loss fully meets the expected loss or is even higher.

#### Analysis

Preliminarily, we applied the Little’s MCAR test to verify if missing data across repeated measurements were at random and then inspected descriptive statistics of sample demographic characteristics as well as of the post-operative weight loss indices.

In accordance with our main aim, we applied a multilevel regression model (Jamovi 2.3.24 solid, GAMLj models; estimation REML) to explore how BSQ scores moderated weight loss trajectory over 24 months after bariatric surgical intervention. Longitudinal data consisted of repeated weight loss observations, i.e., I level variable, nested within 111 patients. Multilevel modelling allowed to inspect individual change over time as well as individual differences in change trajectories, with the fixed intercept representing weight at the initial status and the fixed slope representing the expected individual change over each interval time, and random effects informing on inter-individual differences in baseline levels (random intercept) and rate of change per time unit (random slope). Moreover, multilevel modelling also allowed us to test both longitudinal linear and non-linear trends of weight change, adjusting for between-people differences, i.e., II level variables, i.e., gender, bypass or sleeve intervention, self-reported ideal post-operative weight, and pre-operative BSQ scores. The interaction term Time by BSQ was added in the predictive model to statistically test if BSQ moderated the weight reduction trajectory over time, also against the interaction term Time by pre-operative weight. Quantitative II level variables were centered around the sample mean; random effect for time was included as well, to verify individual differences in longitudinal trajectories of weight loss. MLM analyses were separately run for each of the 3 quantitative indices of weight loss, i.e., %TWL, %EBMIL, and adjusted %EBMIL.

Finally, we applied regression analysis to examine associations between in BSQ scores and weight reduction at different time points, after adjusting for baseline weight, to verify if pre-intervention BSQ scores could anticipate individual differences in weight reduction at a given post-intervention time point, i.e., 6, 12, 18, or 24 months later.

## Results

### Study 1

Missing responses on single BSQ items were in the range 0.3–2.8% of the total responses provided by the present sample; Little’s test of Missing Completely at Random (MCAR) indicated that missing data were completely at random (χ^2^_630_ = 669.6, *p* > 0.10). Accordingly, we used observed BSQ item means to replace missing values.

BSQ items were normally distributed, with skewness < │1.0│and kurtosis < │1.5│, with two only exceptions, i.e., items 26 and 32 which were log-transformed to reduce their deviation from a normal distribution. Furthermore, Kaiser–Meyer–Olkin (KMO) Measure of Sampling Adequacy was equal to 0.97 and Bartlett’s test of sphericity (*p* < 0.001) indicated that the present data sample could be factor-analyzed.

Results from confirmatory factor analysis supported the expected general factor model, with suboptimal CFI (0.86) and TLI (0.85) values, but adequate SRMR (0.053) and RMSEA (0.074, 90% CI 0.070–0.079); standardized estimates were > 0.40, with items 26 and 32 showing factor loadings in the range of 0.20 to 0.30. After inspecting the item residual correlation matrix and modifying the model accordingly (i.e., item 34 covaried with items 2, 18, 33; item 32 with items 25 and 26; item 22 with 6 and 23; item 2 with 4), the model fit further improved (∆χ^2^ = 197, *p* ≤ 0.001), with CFI and TLI ≥ 0.88, SRMR = 0.047, and RMSEA = 0.068. When we tested a two-factor solution in accordance with results from an exploratory factor analysis—which showed fit indices of TLI = 0.90 and RMSEA = 0.061, and r = 0.70 between the two factors—the model comparison between the general factor and the two-factor model did not evidence a significant improvement (∆χ^2^ = 5.0, *p* > 0.05).

Table 1 presents BSQ descriptive statistics which represent preliminary normative values for patients eligible to bariatric intervention. Overall, patients were moderately dissatisfied with their body size, adiposity, and appearance, with significant and substantial gender differences (*p* < 0.001, η^2^ = 0.13). BSQ scores marginally correlated with age (r = − 0.12, *p* < 0.05) and actual BMI (r = − 0.15, *p* ≤ 0.05), and uncorrelated with ideal BMI (r = − 0.10); actual and self-reported ideal BMIs were positively correlated (r = 0.28, *p* ≤ 0.001; for male patients r = 0.42, *p* ≤ 0.001, for female patients r = 0.21, *p* ≤ 0.01), and gender did not significantly moderated the association; on average, the self-reported ideal BMI to reach after intervention was higher among males (28.6 ± 3.4) than females (26.8 ± 4.6; *p* < 0.001, η^2^ = 0.04). Any non-linear associations between the study variables did not emerge as statistically significant.

**Table 1:** Descriptive statistics for Body Shape Questionnaire scores in candidates for bariatric surgical intervention

	*n*	M ± SD	Percentile rank scores				Pearson’s r	
			80	85	90	95	Actual BMI	Ideal BMI
Females	244	128.0 ± 35.5	161	165	171	180	− 0.14*	− 0.05
Males	112	98.9 ± 36.7	132	142	153	169	− 0.03	0.02
Overall sample	358	118.8 ± 38.3	155	162	168	178	− 0.15*	− 0.10

*n* = 340 (68.2% females) for correlations between BSQ scores and actual BMI at interview; *n* = 306 (68.0% females) for correlations between BSQ scores and patients’ self-reported ideal BMI at interview

*p ≤ 0.05, **p ≤ 0.01, ***p ≤ 0.001

### Study 2

*Sample descriptive statistics* Patients’ average age was 43.9 ± 10.3 years and their actual BMI when interviewed as candidates (M = 42.6 ± 5.2) was statistically comparable to actual BMI recorded when they underwent bariatric surgery intervention (M = 43.1 ± 4.6; *p* > 0.05), although on average 11.5 ± 5.3 months passed between interview and intervention; 68.5% of patients were in the range of class III morbid obesity, 20.7% in the range of class II severe obesity, 9% in the range of extreme obesity, and 1.8% in the range of class I obesity. Sixty-eight (61.3%) patients underwent gastric bypass (BMI = 42.40 ± 3.86) and 43 (38.7%) sleeve gastrectomy (BMI = 44.15 ± 5.44) intervention, with the latter patients having a BMI slightly higher compared to the former patients (*p* = 0.05, η^2^ = 0.03).

*Missing values.* Data consisted of 519 observations (93.5% out of expected observations, i.e., weight (or BMI) at intervention, and post-operative weight loss at 6, 12, 18, and 24 months), nested within 111 patients. Specifically, weight (or BMI) was available for all patients at the baseline (intervention), 6 and 12 months after surgery, for 82% at 18 months, and 86% at 24 months. The Little’s MCAR test showed that BMI missing values were completely at random (χ^2^ = 11.58, df = 8, *p* > 0.10).

*Descriptive statistics and longitudinal trajectory of post-intervention weight reduction* Table 2 presents actual BMI and different weight loss indices from surgery to 24 months later, for the present sample. The results suggest a fast weight reduction within the first semester, with an average BMI loss of 12.4 (32.7 kg), 79% of successful weight loss as %EBMIL ≥ 0.50, and an even higher rate for the adjusted %EBMIL index as well as for %TWL; the patients further improved their success across the next 6 months, and mostly maintained, though with some oscillations, their weight during the second post-operative year; the weight reduction indices show consistent results. Overall, the descriptive results in Table 2 indicate a non-linear post-operative trend.

**Table 2:** Descriptive statistics for post-operative weight loss indices across repeated measurement, over 24 months

	6 months	12 months	18 months	24 months
BMI	31.7	29.3	28.7	29.1
Weight loss (Kg)	32.7	39.9	41.2	41.0
%TWL	26.2 (88.2)	31.9 (92.2)	32.8 (95.2)	39.9 (93.3)
%EBMIL	65.5 (79.3)	78.8 (91.9)	81.4 (94.5)	80.3 (87.4)
Adjusted %EBMIL	82.1 (94.6)	99.1 (96.4)	102.0 (97.8)	101.0 (94.7)

In parentheses, percentage of successful patients (i.e., %TWL ≥ 80, %EBMIL < 0.50) is reported; 91 ≤ N ≤ 111. %TWL = percentage of total weight loss (kg), *%EBMIL* = percentage of excess BMI loss, adjusted *%EBMIL* = adjusted percentage of excess BMI loss (Baltazar et al. 2011)

**Fig. 1:**
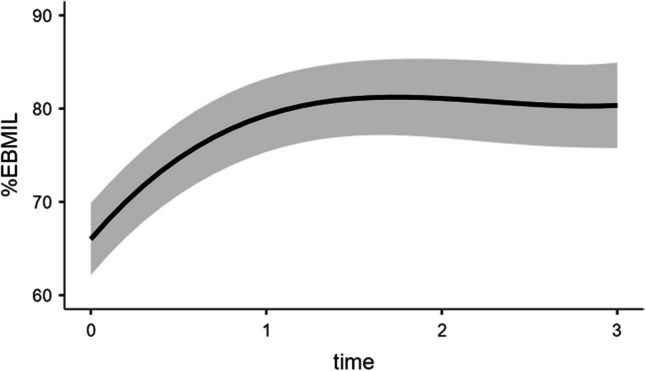
Non-liner trend line and 95% confidence intervals of the model for Excess BMI Loss as percentage. Time or measurement occasion: 0 = 6 months, 1 = 12 months, 2 = 18 months, 3 = 24 months after intervention

Results from multilevel modelling for longitudinal data confirmed that the post-operation weight reduction as %EBMIL trajectory was not linear, but cubic, adjusting for gender (*p* > 0.05), type of intervention (*p* > 0.05), and baseline BMI (b = − 2.03, *p* ≤ 0.001), self-reported ideal BMI (*p* > 0.05) and BSQ scores (*p* > 0.05). Figure 1 illustrates the trend. Specifically, unstandardized estimate b values from the estimated model suggested a linear increasing of %EBMIL across time (b = 22.02, *p* < 0.001)—with a significant random effect (SD = 4.14, *p* < 0.001) demonstrating inter-individual differences in weight reduction over 2 years—but the significant quadratic effect for time (b = − 10.23, *p* < 0.001) indicated that weight reduction became slower and slower, though not constantly across time, and the significant cubic effect (b = 1.49, *p* < 0.005) revealed that the non-linear trajectory had two critical turning points, a local maximum at 16 months and a local minimum at 23 months, that is, patients reached their main success in BMI loss at 16 months, but thereafter they tended to slightly regain weight and lose it again. Results from %TWL and adjusted %EBMIL indices were parallel, and confirmed a non-linear cubic trend over time, with random effect for time being significant as well; type of intervention was significant (b = − 3.18, *p* ≤ 0.05) only when %TWL trajectory was inspected and indicated that patients with sleeve gastrectomy intervention constantly reached a lower percentage of total weight reduction.

**Fig. 2:**
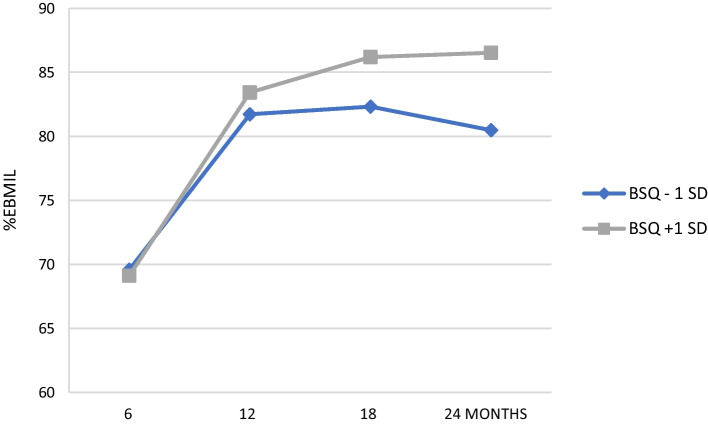
BSQ scores moderate the longitudinal trend of post-operative weight reduction as %EBMIL. Gender and actual BMI at intervention were taken under control; type of intervention, self-reported ideal BMI and time by actual BMI were not included in the current prediction model, because they were not statistically significant (*p* > 0.05)

*Moderation effect of BSQ on weight loss trajectory* To examine the main aim of the present Study, the prediction model included the above predictors of weight loss trajectory, further adding two interaction terms, i.e., time by BSQ scores and time by pre-operative actual BMI (or weight in kg for %TWL). For %EBMIL, the results showed that the former (*b* = 0.03, *p* < 0.05), but not the latter (0.27, *p* > 0.05) interaction term was significant, that is, BSQ but not pre-intervention BMI moderated the post-operative trajectory of weight loss as %EBMIL*,* with lower BSQ scores anticipating progressively less and less successful post-operative weight reduction, as illustrated in Fig. 2. Analysis from %TWL and adjusted %EBMIL yielded the same results, with the time by BSQ score term (*p* ≤ 0.05), but not the time by baseline BMI (*p* > 0.10) being statistically significant; type of intervention or self-reported ideal post-surgery weight did moderate the trajectory neither.

*Correlations between BSQ scores and weight loss indices* Table 3 presents simple correlations and semi-partial correlations (after controlling for pre-intervention weight) between BSQ scores and weight loss over 24 months. Semi-partial correlations evidence that BSQ does not temporally anticipate individual differences in weight or BMI changes, whereas simple correlations warn against possible spurious associations between %EBMIL and BSQ, due to pre-surgery weight as back variable. Conversely, pre-intervention BMI was significantly (*p* ≤ 0.01) correlated with post-surgery outcome, at each follow-up, ranging *r* = − 0.54 for %EBMIL at 6 months (*r* = − 0.37 for adjusted EBMIL) to − 0.41 (*r* = − 0.24 for adjusted %EBMIL) at 24 months; baseline was uncorrelated with post-operative %TWL.

**Table 3:** Semi-partial correlations between BSQ scores and weight reduction at different post-operative time intervals, after controlling for weight at intervention

	6 months	12 months	18 months	24 months
%TWL	0.00 (− 0.02)	0.10 (0.09)	0.05 (0.02)	0.12 (0.10)
%EBMIL	− 0.03 (0.13)	0.07 (0.20*)	0.04 (0.16)	0.10 (0.23*)
Adjusted %EBMIL	− 0.04 (0.07)	0.08 (0.15)	0.04 (0.10)	0.11 (0.18)

In parentheses, simple correlations are reported; 91 ≤ N ≤ 111. %TWL = percentage of total weight loss (kg), *%EBMIL* = percentage of excess BMI loss, adjusted *%EBMIL* = adjusted percentage of excess BMI loss. *p ≤ 0.05

## Discussion

The BSQ is a standard tool routinely applied for evaluation of body image in bariatric surgery candidates [26, 27], but no empirical evidence has been published on its internal consistency and structural validity in such patients. Marzola et al. [44] recently demonstrated the validity of the 34-item BSQ version on a sample of Italian patients with eating disorders, but no further studies are available in the Italian language. The present Study 1 results supported both the internal consistency and the validity for a general factor structure of the questionnaire in candidates for bariatric surgery, thus contributing to extend the validity of this measure among clinical samples, further providing preliminary normative scores for both male and female patients suffering from obesity. Study 1 results also confirm that patients with obesity are considerably more dissatisfied with their body shape, female patients especially, compared to normative data [28–30], although the correlation between BMI and body dissatisfaction is small among patients suffering from obesity rather than of moderate size as in non-clinical samples, thus confirming that the association between body dissatisfaction levels and weight is unclear among patients with obesity yet and needs further investigation [31]. Conversely, the present findings indicate that the higher the baseline BMI, the higher the target weight a patient regards as optimal to reach after intervention, in male patients especially. Overall, the self-reported ideal post-surgery weight remains in the range of overweight and this calls attention on potential consequences of actual post-operative success.

The results from the longitudinal Study 2 showed that the post-operative excess weight loss trajectory has a cubic shape, revealing a positive trend during the first year and a general stagnation during the second year, when weight may still be in the range of obesity and there also is a risk for weight regain. Such a non-linear trend was successfully tested against three different weight loss indices. Clinically, the present findings are consistent with literature [13, 14] and indicate that the second year after intervention represents a critical phase. Hence, bariatric patients need a long-term support to improve their medical conditions.

Our main aim, however, was to explore the hypothesis that body attitudes moderate the post-operative weight reduction trajectory. Study 2 indicated that the trajectory over 2 years was moderated by pre-surgery body shape dissatisfaction, concerns, and feeling of fatness, that is, BSQ accounted for individual variability around the overall weight reduction trajectory. Specifically, our findings suggest that pre-operative lower BSQ scores represent a risk factor for the course of post-surgery weight loss, in accordance with a pathoplasty model linking psychological variables to the course of health-related outcomes [38]. Such a time by body attitudes interaction effect was replicated across the three largely applied weight loss indices of %TWL, %EBMIL, and adjusted %EBMIL. Patients initially less concerned with their body are more at risk of slowly regaining weight 1 year after surgery. Apparently counterintuitive, such a result reflects the weaker and less consistent association between body dissatisfaction and weigh in such a clinical population compared to non-clinical populations, where instead the link is steadily positive [31], although people suffering from obesity generally are markedly dissatisfied with their body image [30, 45], both before and after bariatric intervention, particularly on the long run [46]. Clinically, far from suggesting that a poorer body image is psychologically protective against weight regain, our findings call attention on the need for a better understanding of psychological factors underlying body dissatisfaction and its link with obesity. For example, Dixon et al. [47] revealed that patients in the range of super-obesity place significantly less importance to their appearance compared to patients in the range of obesity, before bariatric surgery. Accordingly, lower BSQ scores might depend on a patient’s resignation towards body appearance and such a feeling should be systematically assessed. Overall, body image plays a key role in bariatric patients, who gain from a systematic psychological support both before and after intervention, when aimed at regaining positive attitudes towards their own body [5].

To date, our study is the first suggesting that body concerns and discomfort shape post-surgery weight loss trends; conversely, body attitudes predicted individual differences in weight reduction neither over shorter or longer time intervals [31, 37]. Such a BSQ moderation effect of weight reduction trajectory remained significant also when the moderation effect of baseline weight was taken under control. Our findings confirm that a lower pre-surgery actual weight as BMI predicts a higher post-operative success for BMI loss indices [17], but also indicate that it does not moderate weight loss course.

Finally, we included patients’ self-rated ideal weight among our studies variables. Generally, poor attention has been given in literature on self-perceived ideal weight after intervention. Our results from Study 1 indicated a positive association between pre-surgery actual and perceived ideal weight, but results from Study 2 showed that such a variable did not contribute significantly in their prediction model of weight loss course. In the future, we might explore how such perceived ideal weight changes across time also in function of actual weight reduction.

### Strengths and limits

Our studies demonstrate the structural validity of the BSQ questionnaire for candidates for bariatric surgery and its external validity against weight loss over 2 years. The results also put attention on the pathoplasty model of post-surgery weight reduction course by evidencing the moderating effect of pre-surgery BSQ scores on the weight loss trajectory. Generally, the two studies contribute to underline the need to support bariatric patients over long time periods and help them preserve and strengthen their success in losing weight and develop a positive body image.

We acknowledge that the present studies have some substantial limits. First, we conducted a monocentric study, with patients involved in our studies being interviewed up to 16 years ago: Generalizability of our findings is limited. Second, BSQ structural validity was tested against the overall sample of patients; further studies are needed to test its multi-group structural validity and its external validity. Third, we mainly focused on the moderating effect of BSQ scores on weight change trajectories across time, but we did not consider other possible models, such as those suggesting that dissatisfaction with body shape predicts weight loss via how a patient copes with dietary and physical exercise prescriptions [35]. Further limits are that we cannot suggest whether body shape attitudes moderate the weight loss trajectories, adjusting for eating habits or psychopathological characteristics. Nor could we inspect post-operative changes in body image concerns or how intra-individual changes in weight and body shape attitudes co-variate over time. Finally, post-operative time measurements also need to cover a longer time than 2 years as we did [13].

### What is already known on this subject?

Pre-surgery weight anticipates bariatric surgery success. People with obesity report body dissatisfaction in the range of subclinical and clinical attention, compared to normative populations. The link between BMI and body dissatisfaction among people with obesity is unclear yet. The heavier the patient with obesity the higher their perceived post-operative ideal weight. Post-operative weight reduction course has a non-linear shape.

### What this study adds?

Psychometric validity of the BSQ questionnaire in candidates for bariatric surgery. Pre-surgery BSQ scores, but not actual or perceived ideal BMI, moderate the post-surgery weight course, with patients initially more dissatisfied with their body shape being more capable of maintaining their post-operative success over 2 years.

## Data Availability

No consent to publicly share data was provided, but data are available for research purposes by emailing the corresponding author.
